# Anxiety and Coping Strategies among Italian-Speaking Physicians: A Comparative Analysis of the Contractually Obligated and Voluntary Care of COVID-19 Patients

**DOI:** 10.3390/healthcare11233044

**Published:** 2023-11-26

**Authors:** Amelia Rizzo, Murat Yıldırım, Izaddin Ahmad Aziz, Zafer Güney Çağış, Łukasz Szarpak, Gabriella Nucera, Aldo Sitibondo, Hicham Khabbache, Francesco Chirico, Behdin Nowrouzi-Kia

**Affiliations:** 1 Department of Clinical and Experimental Medicine, University of Messina, 98122 Messina, Italy; amelia.rizzo@unime.it; 2 Department of Cognitive Sciences, Psychological, Educational, and Cultural Studies, University of Messina, 98122 Messina, Italy; 3 Department of Psychology, Agri Ibrahim Cecen University, Agri 04100, Turkey; muratyildirim@agri.edu.tr; 4 Graduate Studies and Research, Lebanese American University, Beirut 1102-2801, Lebanon; 5 Special Education Department, College of Education, Salahaddin University-Erbil, Erbil 44002, Iraq; izaddin.aziz@su.edu.krd; 6 English Department, College of Education, Bayan University, Erbil 44002, Iraq; 7 Department of Psychology, Mersin University, Mersin 33110, Turkey; zaferguneycagis@gmail.com; 8 Department of Clinical Research and Development, LUXMED Group, 02-676 Warsaw, Poland; lukasz.szarpak@gmail.com; 9 Department of Emergency, Fatebenefratelli Hospital, ASST Fatebenefratelli and Sacco, 20121 Milan, Italy; gabriellanucera@gmail.com; 10 Infectious Diseases Unit, University Hospital “G.Martino”, 98124 Messina, Italy; aldo.sitibondo@gmail.com; 11 Department of Psychology, Faculty of Arts and Human Sciences Fès-Saïss, Sidi Mohamed Ben Abdellah University, Fez 30050, Morocco; hichamcogn@gmail.com; 12 Post-Graduate School of Occupational Health, Università Cattolica del Sacro Cuore, 00168 Rome, Italy; 13 Health Service Department, Italian State Police, Ministry of the Interior, Centro Sanitario Polifunzionale, 00184 Milan, Italy; 14 Department of Occupational Science and Occupational Therapy, University of Toronto, Toronto, ON M5G 1V7, Canada

**Keywords:** occupational health, healthcare workers, volunteering, work issues, COVID-19, physicians

## Abstract

This study aims to explore the differences in the psychological impact of COVID-19 on physicians, specifically those who volunteered or were contractually obligated to provide care for COVID-19 patients. While previous research has predominantly focused on the physical health consequences and risk of exposure for healthcare workers, limited attention has been given to their work conditions. This sample comprised 300 physicians, with 68.0% of them men (mean age = 54.67 years; SD = 12.44; range: 23–73). Participants completed measurements including the State-Trait Anxiety Inventory (STAI), Coping Inventory in Stressful Situations (CISS), and Coronavirus Anxiety Scale (C.A.S.). Pearson’s correlations were conducted to examine the relationships between the variables of interest. This study employed multivariate models to test the differences between work conditions: (a) involvement in COVID-19 patient care, (b) volunteering for COVID-19 patient management, (c) contractual obligation to care for COVID-19 patients, and (d) COVID-19 contraction in the workplace. The results of the multivariate analysis revealed that direct exposure to COVID-19 patients and contractual obligation to care for them significantly predicted state anxiety and dysfunctional coping strategies [Wilks’ Lambda = 0.917 F = 3.254 *p* < 0.001]. In contrast, volunteering or being affected by COVID-19 did not emerge as significant predictors for anxiety or dysfunctional coping strategies. The findings emphasize the importance of addressing the psychological well-being of physicians involved in COVID-19 care and highlight the need for targeted interventions to support their mental and occupational health.

## 1. Introduction

Physicians, including both doctors and nurses, are at the forefront of combating pandemic diseases. The responsibility of physicians to provide treatment during such crises has become increasingly crucial [1,2,3,4]. The COVID-19 pandemic has posed a significant threat to healthcare systems worldwide, pushing intensive care capacities to their limits. However, the moral obligation to prioritize patient well-being above everything else has been tested by an unprecedented situation: the inability to adequately safeguard healthcare personnel with affordable and efficient protective gear, stemming from supply chain breakdowns and shortcomings in institutional planning.

Many studies have focused on the symptomatic aspect and impact of COVID-19 on the medical profession, highlighting the increased risk of exposure to the virus and the physical health consequences of providing care during the pandemic [5,6]. However, few studies have investigated the psychological impact of COVID-19 on physicians, particularly on those who volunteered to provide care to COVID-19 patients [7,8]. For example, Domaradzki and Walkowiak (2021) surveyed 417 medical students, revealing that amidst a decline in traditional volunteering, young individuals’ involvement in volunteering during the health crisis was primarily driven by altruism and an ethical duty to serve their community, physicians, and patients [9]. This volunteering alleviated the strain on the healthcare system and reinforced important medical values such as selflessness and professional solidarity, showcasing the students’ moral commitment to their actions.

Volunteering is essential to public health emergencies, as it provides crucial support to overwhelmed healthcare systems [10,11,12]. Volunteering in the healthcare sector has been vital to the global response to the COVID-19 pandemic [13] and significantly contributed to effectively managing and reducing the transmission of COVID-19 [14]. For example, a study showed that medical students in Canada were found to demonstrate their selflessness by providing aid to physicians and actively volunteering for tasks like aiding in contact tracing. These collective actions hold the potential to mitigate the impact of quarantine or isolation, contributing to the well-being and overall health of healthcare workers [15]. Therefore, healthcare providers are often expected to be essential in responding to the COVID-19 crisis [16]. However, participating in volunteering activities during the COVID-19 pandemic can also give rise to adverse psychological outcomes for physicians, leading to potential challenges to their mental well-being. Studies have found that volunteering during a public health emergency can lead to depression, anxiety, and fatigue [14]. A scoping review has highlighted the impact of the COVID-19 pandemic on the mental well-being of emergency physicians, encompassing volunteers, emergency room doctors, nurses, advanced practitioners, and paramedics. It was found that these individuals, who deliver care during the pandemic, face a heightened susceptibility to various psychological effects, such as anxiety, depression, burnout, sleep disturbances, symptoms of post-traumatic stress disorder, psychological distress, and secondary trauma, as well as stress [17,18,19,20,21].

The contrasting scenario involves physicians who were compelled to treat COVID patients not by their own choice, but due to an organizational decision that reassigned their department to handle COVID-19 cases. During the pandemic’s initial phase, a study [22] evaluated the mental well-being and related factors of healthcare workers attending to patients exposed to COVID-19 in China. The findings revealed that many participants experienced symptoms of depression, anxiety, insomnia, and distress. Notably, nurses, women, frontline workers, and those who resided in Wuhan displayed more pronounced mental health challenges and symptoms of psychological distress. Additionally, the study highlighted that frontline healthcare workers directly involved in treating COVID-19 patients faced an increased risk of encountering emotions like depression, anxiety, sleep difficulties, and distress, compared to their colleagues. Moreover, another study found that physicians experienced sleep disturbances and mental health issues amid the COVID-19 pandemic, with increased non-voluntary night shifts being identified as contributing to these challenges [23]. 

According to Pelkas and Boisseau (2020), the obligation of physicians to provide treatment during a pandemic is determined by the existing laws governing doctor–patient and doctor–employer relationships, in the absence of specific legislative provisions [24]. Bakewell, Pauls, and Migneault (2020) provided insights into the ethical implications concerning the duty to provide care and ensure physician safety during the COVID-19 pandemic. While contractual responsibilities may differ across institutions, there exist non-contractual legal obligations pertaining to medical care during a pandemic and considerations regarding the extent to which physicians can decline work they perceive as unsafe [25]. Moreover, although the obligation to care is firmly established within existing physician–patient relationships, the extent of physicians’ duty to care for individuals who are not yet their patients remains less defined.

This work condition has probably caused psychological consequences that have not yet been estimated. One possible explanation for the adverse psychological consequences of the obligation of healthcare work shift during a pandemic is the stress of providing care in a high-risk environment. Among physicians, those directly engaged in the frontline care of patients face heightened vulnerability compared to their peers. In a study examining the emotional effect of the COVID-19 pandemic on healthcare workers who were directly involved in the care of COVID-19-positive patients, those who personally experienced symptoms, and individuals who felt a lack of access to adequate personal protective measures, exhibited elevated symptoms of depression, anxiety, and stress, compared to their peers who did not face these specific stressors [26]. The factors contributing to these negative psychological effects include an overwhelming workload, extended work hours, inadequate personal protective equipment, sensationalized media coverage, and insufficient support [27]. Another significant factor related to this psychological impact is the infection rate observed among physicians [28]. To our knowledge, no studies have investigated the differences in coping strategies and resources used by physicians who volunteered to provide services and those who were mandated to provide services during the COVID-19 pandemic. This lack of comparative data poses a significant gap in the literature on the psychological aspects of volunteering during a pandemic, and raises questions about the differences in coping strategies and resources used by physicians.

One plausible hypothesis is that the physicians who volunteer to provide services during a pandemic may employ different coping strategies and have different resources available to them than those who were obligated to provide services. Investigating these potential differences may provide valuable insights into the psychological aspects of work conditions during the COVID-19 pandemic. Available evidence shows that during the COVID-19 pandemic, the mental well-being of healthcare workers was influenced by their perception of virus-related threats, personal resources, and their ability to cope. A recent research by Krok et al. (2020) [29] explored how different coping methods, such as tackling issues head-on, handling emotions, and deriving deeper significance, influenced the relationship between how people view COVID-19 risks, the resourcefulness derived from deeper meanings, and mental health outcomes. The results highlighted that those who perceived greater risks experienced lesser mental health benefits, whereas those with more profound, meaning-driven resources experienced enhanced mental wellness. Notably, coping by addressing problems directly and deriving deeper significance were pivotal in linking risk views, profound resources, and mental health. This indicates that perceived threats and individual strengths affect healthcare workers’ mental health indirectly, shaped by their coping approaches. Studies in the literature underscore the relevance of individual coping strategies and available resources in influencing the mental well-being of healthcare workers. Coping strategies are often employed based on how individuals assess stressful situations and their available personal strengths, leading to consequential impacts on well-being and mental health [30]. Furthermore, distinct predictions might emerge for various coping strategies, given that both risk perception and personal resources maintain direct connections with these coping strategies [29]. Therefore, the present study aims to investigate the differences in the psychological impact of COVID-19 on physicians who volunteered and those who were mandated to provide services. We hypothesize that physicians who volunteered to provide services during the pandemic may employ different coping strategies and have different resources available to them than obligated physicians. Furthermore, we expect that these differences may impact the psychological consequences of COVID-19 on physicians. Examining the potential variations between voluntary and obligated physicians can offer important evidence regarding their psychological experiences during the COVID-19 pandemic. By understanding how different coping strategies and resources are used with the demands of their roles, healthcare systems can tailor interventions to better support the mental health and well-being of their personnel. As such, uncovering these dynamics not only enriches our understanding of the pandemic’s psychological impact on Italian-speaking physicians, but also helps to design targeted strategies that foster resilience and psychological health amidst challenging circumstances.

## 2. Materials and Methods

### 2.1. Procedure

In this cross-sectional study, data were collected through the Google Forms © platform, the link was disseminated through mailing lists, social networks, and messaging apps to a convenience sample of Italian-speaking physicians. Participants willingly took part, with their anonymity maintained in alignment with the ethical guidelines outlined in the Declaration of Helsinki for research subjects. Before taking the survey, upon accessing the link, the page presented the informed consent, which explained the study’s objectives and participants’ rights according to the Personal Data Protection Code (EU Regulation 2016/679). Only those who agreed could proceed; otherwise, the link became inaccessible. 

### 2.2. Measures

#### 2.2.1. Sociodemographic Information

To gather sociodemographic information, an ad hoc questionnaire was prepared that consisted of questions regarding personal information (e.g., sex, age, region of residence, and marital status) and work (e.g., type of contract and type of healthcare specialty, COVID-19 pandemic work conditions) (See Table 1).

#### 2.2.2. State-Trait Anxiety Inventory (STAI-Y-1; STAI-Y-2)

The State-Trait Anxiety Inventory (STAI) is commonly used in clinical and research settings to assess adult anxiety levels [31]. It involves separate assessments of state anxiety (temporary symptoms) and trait anxiety (chronic tendencies). The most widely-used version, Form Y, includes 20 items for each type of anxiety. State anxiety items include contrasting statements such as “I am tense” versus “I feel calm.” In contrast, trait anxiety items indicate a person’s general anxiety level or tendencies, for example, “I worry too much over something that doesn’t matter.” Items are rated on a 4-point scale; higher scores indicate greater anxiety levels. In the present study, we used both state and trait anxiety scales, in their Italian version, by Pedrabissi and Santinello (1989) [32], which is suitable for individuals with a sixth-grade reading level or higher. It has excellent internal consistency, with coefficients ranging from 0.86 to 0.95 and a test–retest reliability coefficient over a 2-month interval ranging from 0.65 to 0.75. Studies have also confirmed its construct and concurrent validity. The State-Trait Anxiety Inventory (STAI) was chosen for its widespread recognition in measuring adult anxiety levels, offering separate assessments for temporary and chronic anxiety, making it highly relevant for assessing physicians’ mental states during the COVID-19 pandemic. 

#### 2.2.3. Coronavirus Anxiety Scale (CAS)

The Coronavirus Anxiety Scale (C.A.S.) by Lee et al. (2020) is as a concise mental health assessment tool designed to pinpoint likely instances of maladaptive anxiety related to the COVID-19 pandemic [33]. With its 5 questions, the tool has shown strong consistency and accuracy. The C.A.S. effectively differentiates individuals exhibiting dysfunctional anxiety from those who do not, employing an ideal cut-off score of 9 (boasting 90% sensitivity and 85% specificity). Such outcomes underscore the C.A.S.’s efficacy as a tool for both clinical research and real-world application. The CAS was selected due to its specific focus on COVID-19-related anxiety, validated by its accurate translation into Italian and high consistency. The questionnaire underwent translation from English to Italian and was subsequently translated back, achieving a 98% similarity in phrasing. Upon data analysis, the Cronbach’s alpha coefficient for the scale’s 5 questions stood at 0.83.

#### 2.2.4. Coping Inventory in Stressful Situations (CISS)

The CISS is a self-report instrument for measuring coping, consisting of 48 items originally developed by Endler and Parker (1994) [34]. A version for adults and one for adolescents is available. The assessment comprises 16 items for task-oriented coping, 16 for emotion-oriented coping, and 16 for avoidance-oriented coping. Within the avoidance category, there are two sub-scales: an eight-item distraction sub-scale and a five-item social diversion sub-scale (the remaining three items do not fit into these categories). Participants are prompted to evaluate each statement using a 5-point Likert scale from 1 to 4, where 1 signifies “not at all” and 4 “very much”. The present study used the Italian version of CISS, adapted into the Italian language by Saulo Sirigatti and Cristina Stefanile (2009) [35]. The Coping Inventory in Stressful Situations (CISS) was included to provide a comprehensive understanding of various coping strategies employed by physicians, with its Italian version ensuring cultural relevance and reliability. The text showed good psychometric reliability and validity characteristics, with a Cronbach’s alpha value ranging from 0.71 to 0.86 for each subscale. 

#### 2.2.5. Participants

The sample size was calculated by selecting 5% as the level of precision and 95% as the confidence level, and the population size was inserted as the total number of physicians in Italy (403.454). Results indicate that the appropriate sample size, given the population size and specified combination of precision, confidence, and variability, is 197. In this study, we utilized a convenience sample supplemented by snowball recruitment. Our inclusion criteria focused on ensuring participants were: (1) registered physicians in Italy (2) practicing during pandemic, and (3) aged between 25 and 67 years; while excluding (1) medical students, (2) non-practicing medical professionals, and (3) physicians over 67 years. Table 1 shows the detailed demographic characteristics of the sample.

### 2.3. Statistical Analysis

The data analysis was performed using SPSS 27.0 software (SPSS Inc., Chicago, IL, USA). Statistical significance was set at *p* < 0.05. Descriptive statistics such as mean (M) and standard deviation (S.D.) were used to report continuous variables, while categorical variables were presented as frequencies and percentages. First, the variables’ skewness and kurtosis were tested to check their distributions. Pearson’s correlations were performed to test the relationships between the observed variables. To verify the existence of differences between groups in the psychological adjustment of healthcare workers, a multivariate analysis of variance (MANOVA) was carried out. 

## 3. Results

### 3.1. Correlations between the Variables

Table 2 shows the Pearson’s correlations between the observed variables. Regarding gender, negative relationships emerge with age. Being a female physician is more strongly associated with increased susceptibility to COVID-19 infection, higher state anxiety, higher trait anxiety, greater emotion-focused coping, and higher levels of coronavirus anxiety. Furthermore, being younger is associated with lower levels of both state and trait anxiety, as well as reduced use of emotion-focused coping strategies like avoidance or distraction. Direct involvement in COVID-19 patient care correlates with being a volunteer and contractually obligated to as a physician. Direct exposure also correlates with lower trait anxiety, a problem-focused orientation, and higher levels of avoidance and distraction coping strategies. In this case, a positive relationship with COVID-19 anxiety does not emerge. The condition of being a voluntary physician does not appear to correlate with the psychological variables, nor does having been affected by COVID-19 in the workplace. State anxiety positively correlates with trait anxiety, emotion-focused coping, and COVID-19 anxiety, while negatively correlating with problem-focused coping. Trait anxiety also negatively correlates with problem-focused coping and social distraction coping, while positively correlating with emotion-focused coping and coronavirus anxiety. Coronavirus anxiety, in turn, positively correlates with state and trait anxiety and dysfunctional coping styles, such as emotion-focused coping, distraction coping, and social diversion coping.

### 3.2. MANOVA

The variables’ distributions were between −1 and +1 and may be considered acceptably normal skewness and kurtosis values with non-significant Kolmogorov–Smirnov and Levene tests. To verify the differences between the groups in terms of state anxiety, coping strategies, and the coronavirus anxiety of healthcare workers, a MANOVA was carried out. The independent variables were working directly (or not) with patients affected by COVID-19 (“COVID Patients”) being a volunteer (or not), being contractually obligated (or not), who have contracted COVID-19 during the pandemic (or not); the dependent variables were state anxiety, task-oriented coping, emotion-oriented coping, avoidance-oriented coping, including the sub-components of distraction and social diversion, and COVID-19 anxiety. Table 3 and Table 4 shows the four multivariate models tested. Gender and age were designated as covariates in the analysis.

1. Group based on involvement in the care of COVID-19 patients:
  - Group 1: participants who have not been directly involved in the care of COVID-19 patients (N = 164).
  - Group 2: participants who have been directly involved in the care of COVID-19 patients (N = 136).
2. Group based on volunteering for the management of COVID-19 patients:
  - Group 1: participants who have not volunteered for the management of COVID-19 patients (N = 222).
  - Group 2: participants who have volunteered for the management of COVID-19 patients (N = 78).
3. Group based on a contractual obligation to care for COVID-19 patients:
  - Group 1: participants who do not have a contractual obligation to care for COVID-19 patients (N = 204).
  - Group 2: participants who have a contractual obligation to care for COVID-19 patients (N = 96).
4. Group based on COVID-19 workplace contraction:
  - Group 1: participants who have not contracted COVID-19 (N = 278).
  - Group 2: participants who have contracted COVID-19 (N = 22).

Post hoc statistical powers vary from 100% to 76.7% for smaller groups. The multivariate tests revealed the significant effects of the independent variable of being directly involved in COVID-19 patient care [Wilks’ Lambda = 0.891 F = 4.423 *p* < 0.001]. Being contractually obligated to care for COVID-19 patients also showed a significant effect [Wilks’ Lambda = 0.917 F = 3.254 *p* < 0.001], whilst being a volunteer and being COVID-19infected did not reveal significant effects on anxiety and coping in the model tested.

Physicians directly involved in COVID-19 patients’ care showed slightly lower levels of state and trait anxiety and emotion-oriented coping, while, as regards coping strategies, they scored higher in task-oriented coping, but also in avoidance-oriented and social diversion coping. 

Physicians contractually obligated to care for COVID-19 patients showed slightly higher levels of state and trait anxiety, while, as regards coping strategies, they scored higher in task-oriented coping and higher in coronavirus anxiety.

### 3.3. Gender and Age Effects

Women, compared to men, exhibit significantly higher mean levels of state and trait anxiety (see Table 5). Additionally, the predominant coping style in women is oriented towards managing emotions and anxiety related to COVID-19. Regarding age differences, the groups were divided into four subgroups (see Table 6). Concerning state anxiety, a slight decrease with age can be observed; however, this result is not statistically significant. Similarly, there is a decreasing trend in trait anxiety with age (*p* < 0.01), although this seems to increase in the age group above sixty years. Coping strategies oriented towards managing emotions, problem-solving, and social diversion, also decreased with age, likely reflecting an increase in professional experience. Finally, there are significant differences related to COVID-19 anxiety, which is significantly higher in the age group between 41 and 50 years compared to their younger or older colleagues.

## 4. Discussion

This study examined variations in the psychological impact of COVID-19 on physicians, particularly on those who volunteered or were required to provide care for COVID-19 patients. The study assessed different work conditions: (a) involvement in COVID-19 patient care, (b) voluntary engagement in COVID-19 patient management, (c) a contractual obligation to attend to COVID-19 patients, and (d) the contraction of COVID-19 in the workplace. The results typically supported the hypotheses of this study and are elaborated in the following sections.

The findings indicated that direct involvement in the care of COVID-19 patients is associated with higher levels of state anxiety, suggesting that physicians who directly engage with COVID-19 patients may experience heightened stress and emotional strain levels. Research findings have demonstrated a significant incidence of psychological symptoms [36] and burnout [37,38,39,40] among physicians during the COVID-19 pandemic. They exhibited greater symptoms of mental health problems by exceeding clinical thresholds for state anxiety, psychological distress (i.e., depression, anxiety, and stress), post-traumatic stress symptoms, and a high prevalence of burnout (i.e., emotional exhaustion, depersonalization, and diminished personal accomplishment) [6,20,40,41].

Furthermore, our results showed that physicians tend to employ task-oriented coping strategies, which involve actively addressing the challenges and demands of patient care. However, they also exhibit emotion-oriented, avoidance-oriented, and diversion coping, which may reflect attempts to manage and regulate their emotional responses in the face of challenging circumstances. In line with these findings, earlier research showed that physicians used different coping strategies to regulate their feelings and behaviors [42]. Previous studies typically suggested that a greater emphasis on active, problem-focused coping strategies and minimizing the utilization of emotion- and avoidance-centered coping approaches tend to yield more positive and constructive results in work-related scenarios and everyday life circumstances [34]. Furthermore, amidst the COVID-19 pandemic, a study involving students pursuing healthcare disciplines showed a relatively high prevalence of symptoms of anxiety (27.7%), depression (26.2%), and stress (9.7%), and showed that students encountering psychological distress predominantly engaged in coping mechanisms like emotion-focused coping behaviors (e.g., substance use, venting, self-blame, acceptance, religion) and avoidant coping behaviors (e.g., behavioral disengagement, self-distraction, denial) [43].

Interestingly, no significant association was found between direct patient care and coronavirus anxiety, suggesting that physicians may experience anxiety related to COVID-19 independent of their direct involvement in patient care. Earlier research highlighted that healthcare workers experience significant psychological shifts during their patient care. Among them, nurses are tasked with taking care of their patients and their well-being. Therefore, it is probable that healthcare professionals may exhibit symptoms of anxiety related to the transmission risk of the coronavirus [44,45].

In addition, physicians who have a contractual obligation to care for COVID-19 patients demonstrated higher levels of state anxiety and coronavirus anxiety, highlighting the additional psychological burden imposed by contractual obligations. These individuals may face heightened anxiety due to the perceived responsibility and potential consequences associated with their contractual obligations. However, they also exhibited task-oriented coping strategies, indicating their active engagement in problem-solving and task completion in challenging circumstances [46]. This suggests that physicians with contractual obligations can effectively channel their efforts toward managing the demands and complexities of caring for COVID-19 patients despite their increased anxiety levels. A study involving nursing students, both those who volunteered on the frontline during the COVID-19 pandemic and those who did not, highlighted that participants encountered feelings of uncertainty, anxiety, and fear associated with disease transmission and its management, particularly in the initial stages of the pandemic [47]. Another study on healthcare professionals observed that problem-focused and emotion-focused coping strategies such as acceptance, planning, and active coping emerged as significant protective factors in mitigating psychological distress such as anxiety, depression, and fear associated with COVID-19 [48,49].

Finally, years of experience in the medical field can significantly influence a physician’s response to unprecedented crises like the COVID-19 pandemic, potentially affecting their levels of anxiety and the coping strategies they employ. Likewise, direct exposure to COVID-19 cases presents a significant risk and source of stress, an important aspect to consider in the framework of our study. Medical specializations also play a crucial role, as different specialties might have varying levels of exposure and associated stress; however, their comparison was not possible at this stage of the study.

## 5. Contributions and Implications

This study makes important contributions to understanding the mental health and coping strategies of physicians engaged in COVID-19 patient care by specifically investigating the differences between those who volunteer and those who are contractually obligated. The study provided evidence of the associations between work conditions, anxiety levels, and coping strategies, which facilitate understanding of the psychological impact of COVID-19 on physicians. Regarding the practical implications, this study highlights the importance of considering work-related factors beyond risk exposure when addressing the mental health of physicians. The distinction between voluntary and contractual care highlights the potential divergent psychological experiences within the same profession. As direct exposure and contractual obligation were identified as predictors of heightened anxiety and maladaptive coping strategies, interventions that focus on managing these factors are warranted. Also, our study findings indicate the necessity of tailored support systems for healthcare workers. The findings suggest that addressing physicians’ mental and occupational health requires more than just general interventions. By recognizing the stressors associated with contractual obligation and direct exposure, healthcare institutions can implement targeted strategies to improve positive mental health and adaptive coping strategies in the face of a health crisis. 

## 6. Limitations

In acknowledging the limitations of our study, it is crucial to highlight that the assignment of employees to their respective workplaces and job tasks’ non-random distribution introduce a potential bias in estimating the effects related to workplace well-being measures, as individuals in different roles or environments may experience varying levels of stress, job satisfaction, and overall well-being.

Additionally, the relatively small sample size may result in a reduction of the statistical power making it challenging to detect small but potentially meaningful effects. This limitation is further exacerbated by the presence of significant disparities in the number of participants across various groups, such as those who contracted COVID-19 versus those who did not, and those directly involved in COVID-19 care versus those who were not. The sample presented include potential biases due to an unbalanced gender distribution, a geographical skew towards the south, and a concentration of married physicians, which may impede its representativeness of the broader population. Such imbalances can affect both the statistical power of our results and their generalizability to broader populations. It is also essential to note that our analysis does not reveal causal effects, and any interpretations of the results should be approached with caution. The infrastructure of the healthcare systems and the physician-to-population ratio vary significantly between northern and southern Italy, contributing to disparities in healthcare delivery and access. These infrastructural disparities have implications for the working conditions of medical professionals in different regions of Italy, potentially influencing their workload, stress levels, and overall job satisfaction. During the COVID-19 pandemic, these existing disparities were magnified, as the northern regions, despite having better infrastructure, were hit harder and earlier by the virus, leading to an overwhelming burden on the healthcare system and its workers. In contrast, while the southern regions had fewer cases initially, their less-developed healthcare infrastructure meant that they were potentially less prepared to handle the surge in cases when the virus did spread to these areas.

In conclusion, while our study provides valuable insights into the relationship between workplace assignments, well-being, and various other factors during the COVID-19 pandemic, these findings must be carefully considered. Future research in this area should aim to address these issues, enhancing the robustness and generalizability of the results.

## 7. Conclusions

The present study could have important implications for healthcare systems worldwide. This has been explained by the concepts of ‘compassion fatigue’ and ‘caregiving burden’, as health professions require a high level of relational and empathic engagement [50,51,52]. By identifying the coping strategies and resources physicians use during a pandemic, healthcare organizations can develop targeted interventions to support physicians and mitigate the psychological consequences of pandemics. Several studies have shown that burnout is already a silent epidemic exacerbated by COVID-19 [53,54,55]. Furthermore, by understanding the differences in coping strategies and resources used by volunteers and obligated physicians, healthcare organizations can optimize their use of resources and support physicians more effectively.

## Figures and Tables

**Table 1:** Descriptive statistics of the sample.

Variables	N (%)
Gender	
Male	204 (68.0%)
Female	96 (32.0%)
Age	
<40 years	58 (19.3%)
41–50 years	26 (8.7%)
51–60 years	90 (30.0%)
>60 years	126 (42.0%)
Country	
Northern	54 (18.0%)
Center	12 (4.0%)
South	202 (67.3%)
Islands	32 (10.7%)
Marital Status	
Married	224 (74.7%)
Divorced	8 (2.7%)
Separated	16 (5.3%)
Single	46 (15.3%)
Widowed	6 (2.0%)
Employment Status	
Affiliated MD	82 (27.3%)
Medical manager	176 (58.7%)
Freelancer MD	20 (6.7%)
Specialist in training MD	22 (7.3%)
Specialties	
Medical ^a^	130 (43.3%)
Surgery ^b^	42 (14.1%)
Clinical ^c^	62 (20.6%)
General Practice	16 (5.3%)
None	24 (8.1%)
Other ^d^	26 (8.6%)
Direct COVID-19 care	
Yes	136 (45.3%)
No	164 (54.7%)
Volunteering	
Yes	78 (26.0%)
No	222 (74.0%)
Contractually obligated	
Yes	96 (32.0%)
No	204 (68.0%)
Contracted COVID-19	
Yes	22 (7.3%)
No	278 (92.7%)
Coronavirus anxiety	
Clinical (>9 cut-off point)	10 (3.3%)
Non-clinical (<9 cut-off point)	290 (96.7%)

^a^ Medical Areas: Allergology and Clinical Immunology; Dermatology and Venereology; Hematology; Endocrinology and Metabolic Diseases; Geriatrics; Cardiovascular Diseases; Digestive System Diseases; Respiratory System Diseases; Infectious and Tropical Diseases; Sports Medicine and Exercise; Emergency Medicine; Community Medicine and Primary Care; Internal Medicine; Thermal Medicine; Nephrology; Neurology; Child Neuropsychiatry; Medical Oncology; Pediatrics; Psychiatry; Rheumatology; Nutrition Science. ^b^ Surgical Areas: Cardiovascular Surgery; General Surgery; Maxillofacial Surgery; Pediatric Surgery; Plastic, Reconstructive and Aesthetic Surgery; Thoracic Surgery; Vascular Surgery; Gynecology and Obstetrics; Neurosurgery; Ophthalmology; Orthopedics and Traumatology; Otorhinolaryngology; Urology. ^c^ Clinical Services Areas: Pathological Anatomy; Anesthesia, Resuscitation, Intensive Care, and Pain Therapy; Audiology and Phoniatrics; Clinical Pharmacology and Toxicology; Medical Genetics; Hygiene and Preventive Medicine; Occupational Medicine; Physical and Rehabilitation Medicine; Legal Medicine; Nuclear Medicine; Microbiology and Virology; Clinical Pathology and Clinical Biochemistry; Radiology; Radiotherapy; Health Statistics and Biometry. ^d^ Other: Dentistry, Home Care, Double Specialties, Osteopathy, Psychotherapy.

**Table 2:** Correlations between age, gender, work conditions, coping, state-trait anxiety, and COVID-19 anxiety (N = 300).

Variable	1	2	3	4	5	6	7	8	9	10	11	12	13	14
1. Gender	1													
2. Age	−0.257 **	1												
3.Direct care of COVID-19 patients	0.064	−0.257 **	1											
4. Volunteering	−0.016	−0.086	0.193 **	1										
5. Contractually obligated	0.05	−0.287 **	0.380 **	−0.179 **	1									
6. COVID-19 contracted	0.136 *	−0.126 *	0.052	0.066	0.136 *	1								
7. State anxiety	0.339 **	−0.130 *	−0.095	−0.04	−0.061	0.019	1							
8. Trait anxiety	0.426 **	−0.124 *	−0.130 *	−0.025	−0.082	0.036	0.799 **	1						
9. Task-oriented coping	−0.131 *	−0.101	0.212 **	−0.006	0.162 **	0.064	−0.195 **	−0.225 **	1					
10. Emotion-oriented coping	0.287 **	−0.188 **	−0.052	−0.027	−0.037	0.011	0.508 **	0.668 **	0.065	1				
11. Avoidance-oriented coping	−0.025	−0.151 **	0.213 **	0.101	0.103	0.063	−0.095	−0.112	0.467 **	0.215 **	1			
12. Distraction coping	0.057	−0.094	0.127 *	0.061	0.072	0.074	0.035	0.073	0.310 **	0.370 **	0.877 **	1		
13. Diversion coping	−0.034	−0.285 **	0.242 **	0.125 *	0.160 **	0.082	−0.093	−0.172 **	0.450 **	0.102	0.856 **	0.613 **	1	
14. Coronavirus anxiety	0.279 **	−0.035	0.006	−0.137 *	0.113	0.045	0.471 **	0.490 **	0.066	0.505 **	0.168 **	0.289 **	0.101	1

Note: ** correlation is significant at the 0.01 level (2-tailed); * correlation is significant at the 0.05 level (2-tailed). Columns 1–6 show Spearman’s rho coefficients; columns 7–14 show Person’s correlation coefficients.

**Table 3:** Effect of work conditions on the subjects.

Psychological Variables	Not Directly Involved in COVID-19 Patients’ Care (N = 164)		Directly involved in COVID-19 Patients’ Care (N = 136)		MANOVA		Effect Size
	M	SD	M	SD	F	*p* Value	Hedges’ g
State anxiety	39.96	9.835	38.06	9.401	6.619	0.011 **	0.19
Trait anxiety	38.59	9.293	35.90	8.035	13.663	0.000 **	0.30
Task-oriented coping	40.62	6.384	43.41	5.823	14.234	0.000 **	0.45
Emotion-oriented coping	26.94	9.445	25.41	7.242	5.966	0.015 *	0.17
Avoidance-oriented coping	35.28	7.724	38.66	8.518	10.572	0.001 **	0.41
Distraction coping	13.01	2.804	13.69	3.685	2.731	0.100	0.21
Diversion coping	12.65	3.397	14.22	3.206	11.821	0.001 **	0.47
Coronavirus anxiety	2.61	2.471	2.88	2.883	0.648	0.421	0.11

Note: ** significant at the 0.01 level (2-tailed); * significant at the 0.05 level (2-tailed).

**Table 4:** Effect of contract conditions on the subjects.

Psychological Variables	Not Contractually Obligated to Care for COVID-19 Patients (N = 204)		Contractually Obligated to Care for COVID-19 Patients (N = 96)		MANOVA		Effect Size
	M	SD	M	SD	F	*p* Value	Hedges’ g
State anxiety	38.00	10.347	39.62	7.989	4.563	0.033 *	0.16
Trait anxiety	36.17	9.210	37.93	7.884	5.905	0.016 *	0.19
Task-oriented coping	41.23	6.527	43.29	5.498	6.036	0.015 *	0.33
Emotion-oriented coping	26.47	8.768	25.77	8.050	2.229	0.136	0.08
Avoidance-oriented coping	36.31	8.168	37.88	8.377	1.170	0.280	0.19
Distraction coping	13.24	3.130	13.50	3.488	0.226	0.635	0.08
Diversion coping	13.02	3.378	14.08	3.346	2.967	0.086	0.31
Coronavirus anxiety	2.51	2.522	3.21	2.902	5.087	0.025 *	0.26

Note: * significant at the 0.05 level (2-tailed).

**Table 5:** Gender differences.

Psychological Variables	Male Physicians (N = 204)		Female Physicians (N = 96)		MANOVA		Effect Size
	M	SD	M	SD	F	*p*-Value	Hedges’ g
State anxiety	36.80	9.048	43.98	9.169	33.194	0.000 **	0.79
Trait anxiety	34.87	8.177	42.67	7.806	53.300	0.000 **	0.96
Task-oriented coping	42.25	6.618	41.10	5.449	3.615	0.058	0.18
Emotion-oriented coping	24.78	8.266	29.35	8.311	13.137	0.000 **	0.55
Avoidance-oriented coping	36.98	8.213	36.46	8.373	1.307	0.254	0.06
Distraction coping	13.23	3.283	13.52	3.172	0.258	0.612	0.08
Diversion coping	13.47	3.309	13.13	3.587	4.310	0.039 *	0.09
Coronavirus anxiety	2.19	2.262	3.90	3.066	31.285	0.000 **	0.67

Note: ** significant at the 0.01 level (2-tailed); * significant at the 0.05 level (2-tailed).

**Table 6:** Age differences.

Psychological Variables	>40 Years (N = 58)		41–50 Years (N = 26)		51–60 Years (N = 90)		>60 Years (N = 126)		Kruskal–Wallis Test	
	M	SD	M	SD	M	SD	M	SD	Χ^2^	*p* value
State anxiety	41.90	10.026	40.31	8.983	37.51	9.818	38.70	9.339	7.639	0.054
Trait anxiety	39.90	9.193	39.31	8.749	35.38	8.685	37.22	8.512	10.335	0.016 *
Task-oriented coping	42.24	5.892	43.54	3.455	42.02	7.509	41.29	5.918	4.126	0.248
Emotion-oriented coping	29.24	7.893	29.00	8.899	24.76	6.834	25.37	9.420	17.573	0.001 **
Avoidance-oriented coping	37.90	7.711	36.46	10.527	37.60	8.953	35.83	7.390	4.721	0.193
Distraction coping	13.24	3.481	13.54	4.072	13.56	3.240	13.14	2.966	2.259	0.521
Diversion coping	14.55	2.696	13.23	4.255	13.64	3.799	12.63	3.027	16.443	0.001 **
Coronavirus anxiety	2.31	2.664	4.23	3.581	2.96	2.639	2.46	2.358	9.046	0.029 *

Note: ** significant at the 0.01 level (2-tailed); * significant at the 0.05 level (2-tailed).

## Data Availability

The datasets used and/or analyzed during the current study are available from the corresponding author upon reasonable request.
